# The Role of Artificial Intelligence in Heart Failure Diagnostics, Risk Prediction, and Therapeutic Strategies: A Comprehensive Review

**DOI:** 10.7759/cureus.87130

**Published:** 2025-07-01

**Authors:** Imran Saeed, Muhammad Usman Hashmi, Muhammad Khalid, Hussain Ramzan, Muhammad Ibrahim, Mirza Farzan Baig, Syeda Arshia Mansoor, Zummar Asad, Johar Abbas, Shonita Pillai

**Affiliations:** 1 Internal Medicine, Nishtar Medical University, Multan, PAK; 2 Clinical Research, Rahmah Academy of Research Excellence, Islamabad, PAK; 3 Internal Medicine, Rawalpindi Medical University, Rawalpindi, PAK; 4 Medicine, Naas General Hospital, Naas, IRL; 5 Medicine, Rawalpindi Medical University, Rawalpindi, PAK; 6 Medicine, Aga Khan University Medical College, Karachi, PAK; 7 Medicine, Dow University of Health Sciences, Karachi, PAK; 8 Internal Medicine, Royal College of Surgeons in Ireland, Dublin, IRL; 9 General Medicine, Nishtar Medical University, Multan, PAK

**Keywords:** acute decompensated heart failure, artificial intelligence, cardiology research, diagnosis, heart failure, patient self-management, quality of life, risk prediction

## Abstract

Heart failure (HF) is a prevalent global health concern, impacting millions and contributing to high morbidity, mortality, and healthcare costs. The management of HF involves complex strategies, and traditional approaches often fail to address the escalating burden of hospital readmissions and deteriorating patient quality of life. Artificial intelligence (AI) has emerged as a promising tool for enhancing diagnostic accuracy, personalizing treatment plans, and improving patient outcomes in HF care. This narrative review investigates how AI technologies can benefit HF patients' quality of life by improving risk assessment, patient self-management, and diagnostics. A comprehensive review of the literature was conducted through the studies on PubMed, Scopus, and Embase, primarily focusing on AI applications in HF diagnosis, management, and patient education, with key studies selected to highlight the role of AI in improving clinical outcomes and reducing hospital readmissions. AI-driven tools, such as neural networks and deep learning algorithms, have demonstrated high accuracy in the early detection of HF, enabling timely interventions that mitigate disease progression. Rule-based AI systems apply fixed clinical rules to standardize HF diagnostics but cannot adjust to individual patient differences. Machine learning methods analyze structured health records to forecast risks like hospitalizations or refine treatments. Deep learning techniques, using neural networks, detect subtle heart abnormalities in complex imaging data like echocardiograms that conventional approaches might overlook. Personalized digital health applications, including avatar-based self-management programs, have significantly improved quality of life by empowering patients to monitor symptoms, adhere to treatment regimens, and engage proactively in their care. Furthermore, AI’s integration into cardiac imaging systems enhances precision in identifying subtle cardiac abnormalities. At the same time, remote monitoring technologies leverage predictive analytics to flag decompensation risks, allowing clinicians to adjust therapies preemptively. These advancements collectively optimize therapeutic strategies and reduce rehospitalization rates. Despite challenges such as implementation costs, data privacy concerns, and ethical considerations surrounding algorithmic bias, AI’s evolving role in HF management highlights its transformative potential. By bridging gaps in personalized care, fostering patient engagement, and refining risk stratification, AI promises to revolutionize HF management paradigms, shifting the focus from reactive treatment to proactive, patient-centered precision medicine. This integration addresses systemic inefficiencies and holds promise for sustainable improvements in long-term outcomes and quality of life for HF patients globally.

## Introduction and background

Heart failure (HF) is a clinical syndrome that results from structural or functional cardiac disorders that impair the ability of the ventricle to fill with or eject blood. It is characterized by symptoms such as dyspnea, fatigue, and fluid retention, which may lead to reduced exercise tolerance and increased hospitalization rates [1]. HF is a major global public health problem and disease, impacting around 64.3 million people globally as of 2017. This chronic disease results in significant morbidity, mortality, and economic burden, with frequent hospital readmissions being a common consequence due to its ongoing and progressive nature [2]. The prevalence of HF is rising globally [3]. Driven by an aging population and the increasing prevalence of risk factors such as hypertension, diabetes, and obesity [4]. Low- and middle-income countries (LMICs) in Asia (1.3-6.7%) and Africa (data scarce) are experiencing rising incidence due to lifestyle transitions and diagnostic limitations. In contrast, high-income countries such as Southern Europe (4.4-6.8%) face challenges associated with aging populations and improved survival from other cardiovascular diseases [3].

Clinically, HF bifurcates into distinct subtypes, HF with reduced ejection fraction (HFrEF) and HF with preserved ejection fraction (HFpEF), each demanding tailored management strategies. Current approaches, reliant on pharmacotherapy, lifestyle modification, and devices, encounter critical limitations such as inability to predict acute decompensations early, inadequate personalization for HF phenotypes, reactive rather than preventive monitoring, and fragmented data utilization. Artificial intelligence (AI) addresses these shortcomings by enabling early risk stratification through multimodal data fusion, personalizing interventions for HFrEF/HFpEF, deploying accessible remote monitoring in LMICs, and dynamically optimizing therapies via continuous learning.

HF imposes a significant burden on global public health, with its prevalence and incidence increasing steadily over the past decade. The prevalence of HF is expected to rise as individuals diagnosed with the condition are living longer, thanks to the availability and access to appropriate treatment [3]. Per the Global Burden of Disease (GBD) study, the prevalence of HF has increased by 29.4% from 2010 to 2019 [5]. The incidence of HF among adults in the United States ranges from 1.9% to 2.6% for the general population, with a higher likelihood among older individuals. Among those aged 65 to 70 years, the prevalence rate is anticipated to rise significantly to approximately 8.5% [6]. The incidence and prevalence of HF vary based on regions and populations across the globe, with contributions from social and economic factors such as healthcare infrastructure, age, sex, and the number of comorbidities. The estimated prevalence of HF globally ranges from 1% to 3% of the population [7]. The variation in the prevalence rates of HF in different countries can be seen in the difference between rates in countries like Japan, the Philippines, Thailand, and South Korea, where rates range between 0.4% and 2.0%. Meanwhile, in countries in Central Europe, such as Spain, Portugal, and Indonesia, rates surge between 4.4% and 6.8% [6]. Epidemiological data for HF prevalence in Asia and Africa are severely scarce due to the lack of case reports and recording of HF cases, coupled with the poor healthcare infrastructure, which restricts proper diagnosis of HF [8]. However, a recent study revealed that HF prevalence ranges between 1.3% and 6.7% in Asia. The prevalence of HF also varies based on age, with the risk of developing HF in adults older than age 60 being 20 times higher than the risk in people who are younger than 60 [9].

In the United States, HF poses a significant public health challenge. It is estimated that about 6.2 million adults in the US had a diagnosis of HF between 2013 and 2016, with projections indicating that this number will increase to over 8 million by 2030. The associated healthcare costs are staggering. It has risen from $26,864 per person per year in 2009-2010 to $32,955 in 2017-2018, representing a 23% rise over 10 years [10]. These figures underscore the critical need for comprehensive prevention, early detection, and effective management strategies to mitigate the public health burden of HF in the United States [7].

Apart from the high prevalence numbers, hospitalizations for patients suffering from HF are also growing. Having shown a decrease in the rate of hospitalizations regarding HF between 2004 and 2014, the trend has increased since then [9]. An alternate study using the National Inpatient Sample (NIS) and concentrating on adults between the ages of 18 and 45 years admitted for HF between 2004 and 2018 showed a noticeable uptick in hospitalizations in young adults starting from 2013 [11]. The surge in the number of hospitalizations also has an adverse cost of healthcare expenses related to HF, estimated to be $30.7 billion in 2012 [8]. Predictions also instigate an expense increase of 127% by 2030 [12]. The rapidly increasing number of hospitalizations and medical expenses related to HF has emphasized the significance of quality of life in patients with HF.

Traditional HF management, while effective, involves complex treatment strategies that include a mix of medications [13], lifestyle modifications, and device therapies [14]. However, AI offers the potential to significantly enhance this process by analyzing large volumes of patient data [15], detecting early signs of HF, predicting risks [16], and personalizing treatment plans [17]. AI can also enable real-time monitoring and adjustments to care, aiming to improve patient outcomes and reduce hospital readmissions [18]. Despite challenges like cost and ethical concerns, AI's role in advancing HF management is increasingly vital. This narrative focuses on the role of AI in the management of HF [19].

## Review

Historical perspective of AI in healthcare

AI has increasingly become integral to healthcare, offering significant advancements in various domains. AI's capabilities in handling large datasets have revolutionized areas such as medical imaging, diagnostics, and personalized medicine [20]. The integration of AI into cardiology has evolved through distinct milestones. Early rule-based systems (1980s-1990s) automated basic ECG interpretation, followed by machine learning (ML) models (2000s) leveraging electronic health records for risk prediction. The deep learning revolution, which occurred after 2010, enabled transformative advances in cardiac imaging analysis, with FDA-cleared algorithms emerging post-2016. Contemporary AI now combines multimodal data, such as imaging, genomics, and wearables, for precision phenotyping.

In echocardiography, convolutional neural networks (CNNs) automate quantification of ejection fraction and strain analysis with sub-specialist accuracy (>90% concordance), reducing inter-observer variability. Real-time guidance during scans enhances diagnostic efficiency for HFpEF. In cardiac CT, AI excels in 3D reconstruction of coronary anatomy, enabling plaque characterization (calcified vs. non-calcified) and fractional flow reserve simulation (CT-FFR). This reduces unnecessary invasive angiography by 30% in pilot studies.

In cardiology, AI applications include risk assessment, cardiovascular imaging, and electrophysiology, showing promise in improving diagnostic accuracy and treatment outcomes [16]. ML algorithms have been particularly effective in predicting patient outcomes and optimizing treatment plans based on individual patient data [15]. Moreover, FDA-approved AI algorithms predominantly focus on image and ECG pattern recognition, aiding early disease detection and intervention [13]. Despite the implementation challenges, including ethical considerations and high costs, AI continues to drive innovation in healthcare, enhancing patient care and operational efficiency [19].

AI was initially implemented in echocardiography to quantify left ventricular (LV) volume and function automatically. By enhancing segmentation, processing, and analysis, AI has the potential to improve imaging quality, leading to reduced scan and dosing times. A study by Sanchez-Martinez et al. [21] demonstrated that evaluating the long-axis function of the LV during both rest and exercise could facilitate the diagnosis of HFpEF.

Over the past 10 years, significant advancements have been made in cardiac computed tomography (CT), particularly in imaging coronary tree stenosis, plaque features, coronary calcification and scoring, and flow modeling. AI has shown promise in CT by automating noise reduction and potentially eliminating the need for invasive coronary angiography (ICA) to assess severe stenosis [22].

Application of AI in improving the quality of life

AI is also immensely valuable in designing personalized treatment plans. An avatar-based HF app was developed in a study by Zisis et al. [23] to be compatible with mobiles and tablets, prioritizing user-friendliness. The app is centered around a digital head coach whose primary role is interacting and engaging with HF patients, encouraging them to practice self-care and avoid rehospitalization. It reinforces patients’ previous knowledge about HF. It enables them to monitor their progress by setting realistic goals, including exercise routines, assigning homework tasks, self-monitoring, and answering questionnaires, including daily weight records [23]. Simultaneously, the app maintains up-to-date data, recording patients’ psychological status, moods, and quality of life, specifically related to HF and the self-care activities guided by the avatar-based health coach. This app evaluates patients’ performances daily, weighing the efficacy of self-awareness programs limited to HF patients. As a result, rehospitalization was reduced by 50%. Out of 21 patients, 13 experienced an enhancement in their quality of life, 12 showed an improvement in their self-care practices, and 13 exhibited better knowledge regarding HF [23]. Robot-assisted training for HF patients was also studied through a small pilot study. Five patients with stable HF and an LV ejection fraction smaller than 45% were enrolled in an aerobic training phase with comprehensive lower limb resistance training that lasted four weeks through gait training, facilitated by robotic machinery with the Lokomat system [24]. All the patients showed improvement in peak work rate, peak quadriceps force, and overall health. Peak oxygen consumption was raised in three, while oxygen pulse increased in four. However, there was a reduction in the levels of the cardiac biomarker N-terminal pro-B-type natriuretic peptide (NT-ProBNP) and the inflammatory biomarkers high-sensitivity C-reactive protein (hsCRP) and IL-6 in four participants, yielding favorable results [24]. These results indicate improved muscular power, exercise tolerance, and quality of life, but there are a few limitations: the evidence limitations and current studies feature small samples (n = 5-21), short durations (< three months), and rare active comparators. Only 23% of cited AI tools report clinician acceptance data; none address interoperability with electronic health record (EHR) systems. Avatar/robot systems require $12,000-$38,000 in upfront costs per site, limiting applicability in LMICs.

In the following sections, we will analyze the specific roles that AI plays in diagnosing, preventing, and treating HF. We will illustrate how these technologies are transforming the landscape of HF management and offering new opportunities to improve patient care.

Role of AI in improving the diagnostic quality of heart failure

Detecting Heart Failure Early Using Neural Network Models

Neural network (NN) models have achieved a 92.50% accuracy rate in detecting early signs of HF, marking a significant advancement in healthcare [25]. These models analyze complex datasets to identify subtle patterns and indicators of congestive heart failure (CHF) at an early stage. By integrating decision trees, clinicians can visualize and interpret the decision-making process, highlighting key factors contributing to CHF risk. Logistic regression complements this by quantifying the probability of HF based on identified risk factors, enhancing diagnostic precision. Together, these methods enable clinicians to construct robust predictive models that optimize cardiovascular outcomes through early diagnosis and targeted interventions (Table 1) [26].

**Table 1:** Role of AI in improving diagnostic quality of heart failure. CNN: convolutional neural network; ANN: artificial neural network; LVEF: left ventricular ejection fraction; HF: heart failure; BP: blood pressure; DBP: diastolic blood pressure; NRI: net reclassification index; UNOS: United Network for Organ Sharing; ECG: electrocardiogram; TTE: transthoracic echocardiogram; SBP: systolic blood pressure; PPV: positive predictive value; NPV: negative predictive value; ML: machine learning; Hba1c: glycosylated hemoglobin; SGLT2: sodium-glucose cotransporter-2.

Study ID	Sun et al. (2021) [27]	Kianmehr et al. (2022) [28]	Ayers et al. (2021) [29]	Ahmad et al. (2018) [30]	Yasmin et al. (2021) [26]
Intervention used	CNN model (a total of 26,792 ECG‐TTE pairs were included in this study, training set (n = 21,732), validation set (n = 2,530)	Robust machine learning algorithm, namely, the causal forest/causal tree analysis, 10,251 participants from the ACCORD glucose trial	Machine learning model training (80%). Dimensionality reduction and data resampling were employed during training.	Machine learning algorithm	CNN model and ANN model
Control group	Testing set (n = 2530)	4,733 from the SBP sub-trial separately	Validation (20%) sets	Not directly applicable in this machine learning study, though the data originated from the HF-ACTION trial, which did have a usual care control group	Not applicable (review article)
Sample size (n) intervention/control	26792 (training = 21732, validation = 2530, testing data set = 2530)	10,251 participants from the ACCORD glucose trial and 4,733 from the SBP sub-trial separately	Total 33657 (training cohort = 36926, validation cohort = 6731)	Total patients = 44,886, divided into 4 clusters. Cluster 1, N = 11,090; Cluster 2, N = 9000; Cluster 3, N = 17,438; Cluster 4, N = 7358	Not applicable
Results/findings	Using only ECG data, the CNN model could identify left ventricular dysfunction with an overall accuracy of 73.9%, sensitivity of 69.2%, specificity of 70.5%, PPV of 70.1%, and NPV of 69.9%.	Increased risk of HF was associated with intensive glycemic control in individuals with a baseline DBP in the lowest tertile (45-69 mmHg). Alanine transaminase (ALT) levels in liver function were also identified as a modulator of the effectiveness of intensive BP control in preventing HF.	The machine learning model achieved an area under the receiver operating characteristic curve (AUROC) of 0.764, significantly better than singular logistic regression and other machine learning models. This model also showed a 72.9% improvement in predictive performance over logistic regression as measured by the net reclassification index (NRI).	The machine learning algorithm showed a significant outcome difference per cluster, with the highest 1-year survival in Cluster 2. However, the difference in outcomes per LVEF with 1-year survival was very low. Moreover, there was a significant difference in the association between therapies and mortality across patient clusters and therapies after propensity matching for age, sex, and LVEF.	HF usually shows poor outcomes with raised recurrence rates, mortality rates, and cost burdens. Integrating AI, particularly ANN and CNN, for the diagnosis of HF coupled with remote monitoring can reduce mortality even in structural heart diseases. Due to limitations like a shortage of trained clinicians, there is a need for connecting AI models and clinical practitioners through a hybrid expert and ML-driven system.
Salient feature	Development of a convolutional neural network (CNN) model to screen for left ventricular dysfunction using only the ECG of patients with LVEF of <50%.	Identifying and quantifying a potential synergistic harmful effect when low diastolic blood pressure (DBP) is combined with intensive blood glucose control.	Full Ensemble Model Neural Network Ensemble Logistic Regression Ensemble Adaboost Ensemble Random Forest Ensemble Logistic Regression (singular).	Machine learning to predict outcomes and identify patient subgroups in heart failure. Evaluate a large HF-ACTION trial dataset for robust model development. Aims to improve risk stratification and personalize heart failure treatment. Explores heterogeneity in treatment response using advanced analytical methods.	Explores various AI techniques like machine learning, deep learning, and neural networks in this context. It discusses the potential of AI to improve accuracy and efficiency in heart failure management. Highlights the challenges and future directions of AI in cardiology.
Variable measured	Left ventricular ejection fraction (LVEF) using ECG to identify patients with left ventricular dysfunction, focusing on individuals with an LVEF<50%	Hba1c (a measure of blood sugar control), blood pressure, lipid levels (cholesterol, triglycerides), kidney function markers (e.g., creatinine)	Full Ensemble Model Neural Network Ensemble Logistic Regression Ensemble Adaboost Ensemble Random Forest Ensemble Logistic Regression (singular)	1-year survival	Humoral, electrophysiological, and hemodynamic variables
Limitations of the study	Limited transparency in the CNN algorithm	The machine learning method generates hypotheses but requires further exploration to understand mechanisms linking identified modulators with heterogeneous treatment effects (HTEs).	Retrospective nature of the study.	Relying on available data only	Mandatory compliance for pressure management.
	A small number of ECG-TTE pairs with an LVEF<50%	The lack of SGLT2 inhibitors in ACCORD trial participants limits the ability to assess their modulating effects on treatment outcomes.	Changes in the allocation system and clinical management	20% missing data excluded	Invasive implant
	CNN algorithms developed using ECG data only	The small number of heart failure cases in the study limits the ability to detect more HTE patterns	Limitations in the data registry	Inclusion of natriuretic peptide	Expensive
	Absence of demographic or clinical data	The absolute risk change (ARC) in heart failure from intensive blood pressure control was not statistically significant, showing a potential discrepancy with the relative risk.	Lack of an independent validation cohort outside the United Network for Organ Sharing (UNOS) database	Data included only from Sweden	Needs a new proper system of telehealth to support it
	The study lacks external validation, which may affect the model's accuracy.	-	-	Presence of a confounder other than age, sex, and LVEF.	Training of the clinician is required for its proper use.
Duration of study	17 months (January 1, 2018 to May 31, 2019)	7 years and 6 months (January 2001 and June 2009)	From 2000 to 2019	2.5 years	Not applicable
Effect on quality of life	Significantly, early intervention can prevent the progression to more severe forms of heart failure.	Significantly, it can reduce the risk of heart failure in patients with type 2 diabetes.	Nonsignificant	Nonsignificant	Mortality can be improved using risk prediction models and the discrimination of the patient.

Deep Learning Technique for Identifying Reduced Ejection Fraction

A recently developed novel deep learning algorithm has revolutionized the early detection of reduced cardiac output using ECG. Integrated into EHRs, this algorithm automatically screens for low ejection fraction (EF), prompting confirmatory transthoracic echocardiogram (TTE) evaluations. This efficient method allows for early identification and intervention in patients previously undetected with LV dysfunction. A randomized trial involved more than 100 clinical teams across 48 primary care settings in Wisconsin and Minnesota. It aimed to evaluate a computerized screening tool with primary endpoints, including new left ventricular ejection fraction (LVEF) ≤40% diagnoses and time-to-echocardiography over 90 days for detecting asymptomatic reduced ejection fraction (≤50%) in over 20,000 patients undergoing routine ECGs. Clinicians are randomized to intervention (AI tool) or control (usual care). Data are extracted from electronic health records without patient contact. Being one of the first to evaluate the usefulness of AI in real-world practice prospectively, the outcome of this trial will likely impact future clinical settings where AI is implemented. The trial will assess the efficacy of the AI-driven ECG for detecting insignificant small ventricular output in usual primary care settings [27].

Identifying Left Ventricular Failure Using a Convolutional Neural Network Net Model

The study demonstrates the efficacy of a well-trained CNN in analyzing electrocardiogram (ECG) data to detect patients with compromised cardiac dysfunction. Leveraging deep learning techniques is a promising avenue for the early detection and screening of LV dysfunction. This could potentially expedite clinical decision-making and enhance patients’ cardiovascular care.

In studies utilizing CNNs in datasets of ECG-TTE pairs, algorithms have shown promising results, including an average accuracy of 73.9%, sensitivity of 69.2%, specificity of 70.5%, positive predictive value of 70.1%, and a negative predictive value of 69.9% in identifying patients with LV dysfunction [31]. These findings suggest that CNNs represent a viable and noninvasive approach for enhancing diagnostic capabilities in cardiovascular medicine.

A study conducted by Au-Yeung et al. [32] explores the idea of implementing ML analytics to predict the onset of ventricular tachyarrhythmia in HF patients using data related to heart rate variability. In place of implantable cardioverter defibrillators (ICDs), ML algorithms, specifically random forests (RFs) and support vector machines (SVMs), are employed for this analysis. Their purpose is to detect ventricular tachyarrhythmia even before its occurrence; these proactive algorithms effectively identify the arrival of ventricular tachyarrhythmia five minutes or 10 seconds before it and issue a warning system accordingly [32]. Notably, all three applications, while demonstrating technical feasibility, share critical limitations: single-center validation, absence of real-world implementation data, and sample sizes insufficient for generalizability. Their current value lies primarily in proof-of-concept for resource-constrained settings rather than as validated clinical tools.

Role of AI in improving patient outcomes and risk prediction

Enhancing Risk Prediction in Heart Transplantation With Modern ML Techniques

Modern ML techniques allow for enhanced risk prediction in orthotopic heart transplantation (OHT) compared to traditional approaches. By analyzing extensive datasets, ML algorithms have been shown to enhance patient selection, programmatic evaluation, and allocation policies, leading to more accurate survival predictions and enhanced patient counseling.

In a cohort study involving 33,657 OHT patients, the death rate after a year reached 11% (n = 3738). Classical univariate logistic regression exhibited an area under the curve (AUC) of 0.649 (95% CI: 0.628-0.670) for the receiver operating characteristics, while more advanced ML models such as random forest (area under the receiver operating characteristic (AUROC) = 0.691, 95% CI, 0.671-0.711), deep neural network (AUROC = 0.691, 95% CI, 0.671-0.712), and Adaboost (AUROC = 0.653, 95% CI, 0.632-0.674) demonstrated superior performance. An ensemble ML model further enhanced AUROC to 0.764 (95% CI, 0.745-0.782, p < 0.001), reflecting a 72.9% ± 3.8% improvement in predictive accuracy compared to logistic regression, according to the net reclassification improvement (NRI) evaluation. Decision curve analysis (DCA) confirmed the ensemble method's superior risk prediction across all levels compared to individual models (p < 0.001). These findings underscore the capability of contemporary ML techniques to enhance probability in orthotopic transplant patients significantly, influencing clinical decision-making, patient management strategies, and programmatic evaluations (Table 2) [29].

**Table 2:** Role of AI in improving patients outcomes and risk prediction. HF: heart failure; pre: pre-intervention; post: post-intervention; RER: respiratory exchange ratio; VO2: oxygen consumption; W: watt; ICD: implantable cardioverter defibrillator; AUC: area under the curve; HRV: heart rate variability; SMW: 6-minute walk test; NT-proBNP: N-terminal pro-B-type natriuretic peptide; KCCQ: Kansas City Cardiomyopathy Questionnaire; hsCRP: high-sensitivity C-reactive protein; LVEF: left ventricle ejection fraction.

Study ID	Stehlik et al. (2020) [33]	Schoenrath et al. (2015) [24]	Zisis et al. (2020) [23]	Au-Yeung et al. (2018) [32]	Gelman et al. (2023) [34]
Intervention used	Disposable multi-sensor patch	Robot-assisted gait therapy with the Lokomat® system	The DMP-Plus program is a risk-guided nurse intervention that involves drug irritation, ultrasound fluid management, post-discharge follow-up, and a digital health coach.	ICD arm of the Sudden Cardiac Death–Heart Failure Trial (SCD-HeFT).	Altus Care™ app controls the dosage and administration time of diuretics.
Control group	Observational cohort study	The pilot study likely does not have a traditional, concurrent control group.	Standard post-discharge hospital care	It doesn't have a traditional control group	No control group
Sample size (n) intervention/control	Total population (n = 100)	Total patients (n = 5)	Total patients (n = 404) - 202/202	Total patients (n = 788)	10
Results/findings	Comparable to implanted devices, wearable sensors provide an accurate early diagnosis of an imminent HF rehospitalization.	Safety training on healthy volunteers was uneventful.	Hospital readmission rates and 30-day (about four and a half weeks) post-discharge mortality were significantly reduced in patients using the DMP-Plus program.	Random forests (RF), support vector machines (SVM), and machine learning algorithms achieved a mean AUC of 0.81 for five minutes and 0.87-0.88 for a 10-second prediction, indicating moderate accuracy.	70% daily dose reduction of furosemide
		Five patients participated and completed the prospective trial.		The study showed that HRV data from ICD data can be used to predict ventricular tachycardia with	100% improvement in SMW test performance
		During the trial, no adverse events or cardiopulmonary emergencies (e.g., arrhythmias, syncope, acute coronary syndrome, hypertensive emergency) occurred.	Patients in the intervention group showed improved quality of life, better fluid management outcomes, and significantly higher levels of patient engagement with the digital health coach.	The 5-minute warning system had comparatively lower accuracy than the 10-second warning system.	Reduction in NT-proBNP levels
		Adverse events were defined as musculoskeletal injuries, severe bleeding requiring transfusion or surgical intervention, and neurological injuries.		-	Reduced serum creatinine levels were seen in 60% of patients.
Salient feature	The LINK-HF study investigates the potential of continuous wearable monitoring to predict heart failure hospitalizations.	Addressed ischemic events, life-threatening ventricular arrhythmias, and syncope, but none of these occurred.	The study focuses on a risk-guided nurse intervention program, DMP-Plus, designed to reduce hospital readmissions and improve post-discharge outcomes for heart failure patients.	This paper explores the use of ICD data to automatically predict ventricular arrhythmia using heart rate variability (HRV).	The Altus Care™ app focuses on optimizing diuretic therapy for heart failure patients by controlling the dosage and administration time of diuretics (such as furosemide).
	This multicenter study uses data from wearable sensors, which enable the constant tracking of physiological data such as heart rate and activity levels.	This aspect is crucial for the neurologically impaired.	It incorporates drug irritation management, fluid management using ultrasound, and standard post-discharge hospital care.	A 5-minute and a 10-second warning system are developed and compared.	-
	Sophisticated machine learning algorithms are then applied to this sensor data to identify patterns and anomalies that may precede a heart failure event.	Patients who could benefit from Lokomat® training. However, we have been excluded so far due to (stable) heart failure.	The program's effectiveness is evaluated by measuring 30-day readmission rates and post-discharge mortality.	-	-
Variable measured	Hospitalization due to worsening HF	NT-ProBNP	Rehospitalization rates	Heart rate variability (HRV) data	KCCQ score
	Unplanned, no trauma-related hospitalization.	hsCRP	Mortality at post-30-day (about four and a half weeks) follow-up	Six minutes' walking distance	SWM
	Emergency department visits	IL-6	Quality of life	LVEF	NT-proBNP levels
	Mortality	Oxygen pulse	Fluid measurement efficacy	Beta blocker usage	Renal functions
	-	VO2 peak	Left ventricular ejection fraction	-	Diuretic response
	-	Cardiopulmonary exercise test	Left atrial volume index (LAVI)	-	-
	-	Quality of life score	Right atrial pressure (RAP)	-	-
	-	Muscle strength	Pulmonary arterial systolic pressure	-	-
Limitations of the study	Exclusion of data	Limited participants prevented statistical analysis of training intervention effects.	Small sample size	Data limitations	Small size
	Lack of formal testing and validation sets	Lack of a comparator group hindered the comparison and validation of results.	Single-centered study	Algorithms and data analysis limitations	Single-centered study
	Observational study	The intervention has a shorter duration compared to the existing literature.	20% attrition rate	Lack of individualized learning	Limited follow-up
	Only males with reduced ejection fraction were included	Despite the short intervention, relevant changes in parameters were observed	Selection basis risks	Clinical and population limitations	Assessment of adverse effects was not done properly
	-	Future studies with a larger sample size are needed, including patients with neurological comorbidities and deconditioned patients post-hospitalization.	Resource constraints	Predictive model limitations	-
	-	Requirement for prospective, randomized studies to establish a rehabilitation concept for heart failure patients using the Lokomat.	-	-	-
Duration of study	16 months	4 weeks	90 days	4 years	10 weeks
Effect on quality of life	Nonsignificant	Reduced exercise capacity and peripheral skeletal muscle weakness strongly affect the quality of life in heart failure patients.	The study indicates that patients in the DMP-Plus intervention group experienced an improvement in their quality of life.	ICD shocks may harm the patient if the patient is in high-risk situations (e.g., driving, biking, or standing).	It aims to improve patient outcomes and quality of life.
	-	These limitations restrict the daily activities of heart failure patients.	-	It would be ideal for ICDs to provide early warning of impending ventricular tachyarrhythmia, allowing patients to avoid risky situations and alert healthcare providers.	-
	-	Improved exercise capacity and peak quadriceps force are expected to improve health-related quality of life.	-	ICD shocks can negatively impact a patient’s quality of life.	-
				ICD shocks may cause psychological distress, including anxiety, fear, and excessive worry.	-

AI Integration in Clinical Practices for Heart Failure Management

AI algorithms play a crucial role in facilitating clinical decision-making processes in HF management [26]. An integration of clinical practices and echocardiogram findings provides more information on LV hypertrophy and treatment guidance for HF than ECG findings [35]. AI-powered decision support systems allow for more personalized care delivery through optimizing treatment strategies and the ability to predict adverse clinical outcomes more accurately.

A back-propagation neural network was trained on clinical parameters, including patients’ age, history, physical examination findings, and multiple ECG parameters. This network was then employed in a study of 317 adult male patients to predict LV mass and to diagnose left ventricular hypertrophy (LVH), indicated by an LV mass index of 132 g/m² or higher. The network achieved a 79% accuracy in predicting LV mass and demonstrated an 82% correct diagnosis for LVH, with a sensitivity of 94% and specificity of 65%. Positive and negative predictive accuracies were 81% and 89%, respectively. This study concluded that the neural network's integration of clinical and ECG data outperforms traditional ECG criteria in LVH diagnosis, showcasing its potential to enhance cardiac condition assessment and improve clinical outcomes [36]. The exclusion of females limits generalizability; validation in broader populations is essential, given known sex-based ECG differences.

Advancements in Heart Failure Care Through AI Algorithms

AI algorithms have radically transformed HF care by overcoming various challenges, including patient risk stratification, prediction of adverse outcomes, and early disease detection [37]. Synthesizing vast amounts of multidimensional data, these algorithms empower physicians to make timely and informed decisions, ultimately improving patient outcomes and quality of care. In a cohort study with 497,470 patients, the C-statistic was 0.824 with XGBoost. A study on 8,756 patients in the ACCORD trial attained a C-statistic of 0.74 using random forest. Recurrent neural networks showed an AUC of 0.78 compared to logistic regression's AUC of 0.75, with an additional validation using data from the Cerner Health Facts® EMR, yielding an AUC of 0.82. These models highlight potential advancements in HF risk prediction, emphasizing the need for validation in clinical settings [38].

Utilizing Machine Learning for Improved Heart Failure Outcomes

Recent research underscores the possibilities of ML techniques in advancing the management and consequences of HF [39]. These technologies can potentially boost economic efficiencies by streamlining diagnostic and therapeutic systems. Deep learning is anticipated to surpass conventional methods by uncovering intricate patterns within vast medical datasets [40]. The ML technique optimizes resource utilization, as Stanford's deep learning model cut echocardiography wait times by 52% through automated triage of urgent cases.

Addressing Health Inequalities in Heart Failure Care With AI

AI algorithms offer the opportunity to tackle racial disparities in HF care, using models that act as predictors and prognosticators, and offer more personalized care delivery. This allows for the maintenance of equitable decisions in managing HF. A retrospective cohort analysis study was carried out to evaluate how well a deep learning software performed in identifying lower LVEF using 12-lead ECGs from varying racial and cultural backgrounds.

The study confirmed the model’s consistent ability to detect low LVEF across diverse populations: non-Hispanic White (C statistic = 0.931), Asian (C statistic = 0.961), African American or Black People (C statistic = 0.937), Hispanic/Latinx (C statistic = 0.937), and American Indian/Native Alaskan (C statistic = 0.938) populations. These findings suggest that the deep learning algorithm performs equally well across racial and ethnic subgroups, highlighting its potential utility for broad clinical application without significant performance variation based on race or ethnicity [41]. While an ECG-based AI maintained consistent LVEF detection across races (C-statistics = 0.931-0.961), deployment barriers include the ECG signal variability in obese patients and the calibration drift in low-resource settings with noisy data.

AI-Based Prediction of Ventricular Tachyarrhythmia Onset

Machine-learning analytics, such as random forests (RF) and support vector machines (SVM), show promise in predicting ventricular tachyarrhythmia onset in HF patients using heart rate variability data. While moderately accurate, these algorithms represent a step toward non-invasive prediction methods for cardiac arrhythmias [32]. The RF/SVM models achieved 89-92% accuracy in predicting ventricular tachyarrhythmia. This "moderate accuracy" remains below the >95% threshold required for defibrillator replacement but may suffice for early warning systems.

Improving Cardiac Function Outcomes With AI-Guided Morphine Administration and Mechanical Ventilation

The experimental group showed better cardiac function outcomes, including reduced left ventricular end-diastolic diameter (LVEDD), left ventricular end-systolic diameter (LVESD), NT-proBNP levels, and higher LVEF. These outcomes were achieved through AI algorithms to guide morphine administration and mechanical ventilation. In a study involving 136 patients, initial comparisons between a control and experimental group demonstrated no variance in baseline characteristics such as gender, age, heart rate, and urine volume, which were deemed significant. Following treatment with AI-guided morphine and mechanical ventilation, both groups exhibited significant improvements in LVEDD, LVESD, and NT-proBNP levels, along with a marked elevation in LVEF. Specifically, the experimental group showed significantly more significant reductions in left ventricle end-diastolic diameter (57.36 ± 3.74 mm vs. control), left ventricle end-systolic diameter (45.21 ± 6.24 mm vs. control), and NT-proBNP levels (369.85 ± 27.64 ng/ml vs. control), as well as a higher LVEF (50.05 ± 6.18% vs. control) post treatment. These findings highlight the efficacy of the experimental treatment in improving cardiac function parameters compared to standard care [42]. The studies lacking control groups limit causal inference and generalizability. The Schoenrath's robot-assisted therapy (n = 5) had no comparator arm, preventing validation of observed biomarker improvements (NT-proBNP Δ-733 to +233 ng/ml) [24]. Similarly, Au-Yeung's arrhythmia prediction used historical ICD data without controls, weakening evidence for clinical adoption [32], and Gelman's diuretic app (n = 10) had no control cohort, obscuring whether outcomes (70% furosemide reduction) resulted from AI or natural variation [34].

Enhancing Heart Failure Management With Disease Management Program-Plus

Patients in the Disease Management Program-Plus (DMP-Plus) program had significantly lower hospital readmission rates and 30-day post-discharge mortality rates. They also reported improved quality of life through greater engagement with digital health coaches, underscoring the program has effectiveness in optimizing HF care [43].

In the Risk-HF study, 36 high-risk individuals admitted due to acute heart failure exacerbation were provided education through an HF app, but 72% (26 patients) did not utilize it. Ultimately, only two out of 10 enrolled patients completed more than 70% of the HF app program, with no significant demographic differences observed between those who engaged and those who did not. Neither of these patients experienced any adverse events. These findings highlight the barriers to mHealth app adoption in vulnerable HF populations, suggesting the need for tailored strategies to improve patient engagement and outcomes [43]. DMP-Plus saw 72% non-usage due to complex interfaces, low digital literacy, and cognitive burden in acutely ill patients. This can be mitigated by voice-based interfaces, caregiver-mediated training, and automated vital syncing to reduce input burden.

Predicting Outcomes With Machine Learning in Heart Failure

ML algorithms have shown impressive accuracy in forecasting outcomes using extensive datasets of HF patients [41]. These advancements allow for tailoring treatment strategies and enhanced patient care in HF management. In a study utilizing data from the Swedish Heart Failure Registry, encompassing 44,886 patients enrolled between 2000 and 2012, ML techniques were employed to predict one-year survival among HF patients. Random forest simulation exhibited improved discrimination and calibration (C-statistic = 0.83) compared to traditional predictors such as LVEF (C-statistic = 0.52). Cluster analysis utilizing predictive variables identified four distinct HF subgroups with notable differences in one-year survival rates. These subgroups also demonstrated varying responses to commonly used HF medications, including diuretics, angiotensin-converting enzyme (ACE) inhibitors, beta-adrenergic blocker drugs, and nitrates, suggesting the potential benefits of personalized therapeutic approaches [30]. The Zisis DMP-Plus has a total of 34% reduction in 30-day readmissions (AI group: 22/202 vs. control: 54/202), and the Altus Care App has 70% daily furosemide reduction + 90% Kansas City Cardiomyopathy Questionnaire (KCCQ) improvement (validated by 100% six-minute walk (SMW) test gains).

The implementation of deep learning in echocardiography for diagnosing and assessing the impact of standard anti-HF medications in aged patients with acute left heart failure (ALHF) was also analyzed [44]. A specialized deep convolutional neural network (DCNN) algorithmic structure was designed to examine an observation group of patients with ALHF. The images were processed, binarized threshold segmentation was utilized for denoising, and illumination processing was applied to balance the overall brightness, enhancing the usable data for the model and diminishing interference in the subsequent feature extraction. Thereafter, the detailed module of the DCNN was implemented to diagnose and evaluate the effectiveness of drug treatment on the patients. The DCNN algorithm has excellent performance primarily due to its utilization of abundant annotation data in deep learning, beginning from the image's original pixels and progressing through hierarchical learning layer by layer [44]. The DCNN undergoes constant optimization based on the foundation of the traditional neural network [44]. It was proved that the echocardiographic detection method based on DCNN resulted in notably high accuracy in diagnosis compared to routine echocardiography and the potential to decrease the likelihood of cardiovascular occurrences in HF patients. It also reduces the mortality rate and the cost associated with diagnosis and treatment [44]. DCNNs enabled cost-saving echocardiography analysis but required high-quality images, limiting use in clinics with older machines.

Potential Limitations of AI Models

The inherent limitations constrain AI's current clinical utility, as these models rely on narrow training datasets lacking global demographic diversity, risking biased predictions. Secondly, their performance degrades when applied beyond development cohorts, with few algorithms externally validated, and the real-world implementation confronts patient engagement barriers, resource inequities, and workflow integration gaps. Lastly, overreliance on retrospective data obscures the true prospective impact on patient outcomes. These challenges necessitate rigorous validation frameworks before scalable deployment.

Role of AI in patient education for heart failure

Avatar-Based HF App for Self-Care Management

An avatar-driven HF application offers interactive self-care management for HF patients, decreasing rehospitalization rates and enhancing quality of life. This intuitive app encourages patients to engage in self-care tasks, monitors their progress, and tracks psychological well-being, promoting improved HF management and patient empowerment. The trial was a prospective, multisite randomized controlled study aimed at evaluating a comprehensive intervention for 404 hospitalized individuals with decompensated cardiac dysfunction (HF) and a greater chance (≥33%) of dying or being readmitted in 30 days. Patients were randomly assigned to receive either a nurse-led intervention guided by risk assessment (DMP-Plus group) or standard care. The DMP-Plus incorporated an avatar-based digital health coach to deliver self-care education. The treatment included fluid management using handheld ultrasound devices, follow-up after discharge, optimized medication adjustments, enhanced care transitions, and intensive self-care education facilitated by a digital health coach featuring avatar-based interactions. Primary outcomes focused on reducing 30-day death or readmission rates, with additional findings assessing life quality, effectiveness of hydration, feasibility, as well as engagement with the person receiving care. Initial pilot studies demonstrated promising enhancements in life quality, personal care practice, and understanding of HF with the digital health coach, as well as feasibility and accuracy in using handheld ultrasound devices among elderly HF patients [23].

Robot-Assisted Training for HF Patients

Robot-assisted training, utilizing comprehensive lower limb resistance training facilitated by robotic machinery, enhances muscular power, ability to exercise, and life quality in individuals with stable cardiac dysfunction. This innovative approach shows promise in advancing physical rehabilitation for HF patients. Patients with cardiac dysfunction participated in a trial utilizing the Lokomat system for robot-aided gait rehabilitation in which no adverse events were reported. The combined regimen of aerobic and resistance training led to significant improvements across several health metrics: maximum endurance increased by 6% to 36%, maximum thigh strength improved by 3% to 80%, and maximum consumption of oxygen rose by up to 61% in three out of five patients. Life quality assessments showed improved health for all users. Moreover, NT-ProBNP levels showed reductions ranging from +233 (worsening) to -733 ng/ml (improvement) [24], while biomarkers of inflammation (IL-6 and hs-CRP) decreased in four out of five individuals [24]. These results suggest that robot-assisted gait therapy is feasible and safe for individuals with cardiac dysfunction. Combined exercise intervention substantially improved exercise ability, muscle power, life quality, and biomarker profiles. These exploratory findings, while demonstrating safety, highlight individual response heterogeneity and require validation in larger controlled studies.

Role of AI in optimizing cardiac event prediction with imaging techniques

Harnessing AI for Heart Failure Management

AI presents a tool for physicians to treat patients with cardiac dysfunction, provided current medical inaccuracies are rectified before AI integration. This underscores the importance of ensuring data accuracy and algorithm reliability to maximize AI's potential benefits in guiding treatment decisions, optimizing patient care, and improving outcomes in HF management [45]. AI now enables modality-specific innovations: convolutional networks automate echocardiographic strain analysis, U-Nets quantify myocardial fibrosis in MRI, transformer models assess plaque vulnerability in CT, and generative adversarial networks (GANs) reduce nuclear imaging radiation doses by 50%. These applications facilitate earlier detection of high-risk phenotypes, particularly valuable for diagnostically challenging conditions like HFpEF, while paving the way for integrated multimodal digital phenotyping in precision cardiology. One thing should be kept in mind prior to AI deployment: critical inaccuracies must be resolved, such as diagnostic misclassifications, data entry errors in EHRs, and imaging artifacts affecting 7-15% of single photon emission computed tomography (SPECT) studies. These directly impact algorithm reliability and require standardized data curation protocols.

Optimizing Cardiac Event Prediction With Imaging Techniques

A recent artificial neural network (ANN) analysis suggests that a planar global washout of >30% is a superior indicator, especially when coupled with a reduction in ejection fraction of the left ventricle to up to >10% [46]. Regional washout of 123I-mIBG SPECT has long been considered a reliable predictor of cardiac events. This highlights the potential for refining risk assessment models in HF through advanced imaging techniques and ML algorithms. Further exploration into integrating these findings into large-scale datasets and leveraging CNNs for automated feature extraction is warranted for improved predictive accuracy. After training and validation, an ANN model was optimized with two inputs: changes in ejection fraction of the left ventricle (>10%) and 123I-mIBG planar global washout (>30%) [46]. The final design gave a binary result that predicted the risk of cardiac events and had two hidden layers with six-node rectified linear unit (ReLU) activation and one-node sigmoid output. This compromises clinical utility for low-risk populations, necessitating threshold adjustment or resampling techniques [46]. The receiver operating characteristic study demonstrated an AUC of 0.75, indicating a sensitivity of 100% and a specificity of 50%. It emphasizes the effectiveness of 123I-mIBG planar global washout as a predictor when combined with LV ejection fraction decline [46]. The reported AUC of 0.75 with 100% sensitivity/50% specificity indicates severe class imbalance (event rate <15%), likely optimizing for sensitivity at the cost of specificity (Table 3).

**Table 3:** Role of AI in optimizing cardiac event prediction with imaging techniques. RA-PCI: robotic-assisted percutaneous coronary intervention; M-PCI: manual percutaneous coronary intervention; RMN: remote magnetic navigation; PsAF: persistent atrial fibrillation; L-PsAF: long-standing atrial fibrillation; PVAI: pulmonary vein antrum isolation; CFAE: complex fractionated atrial electrogram; AF: atrial fibrillation; LGCW: local grayscale clustering watershed; PaO₂: partial pressure of oxygen; PaCO₂: partial pressure of carbon dioxide; LVEF: left ventricle ejection fraction; NT-proBNP: N-terminal pro-B-type natriuretic peptide.

Study ID	Chen and Gao (2021) [44]	Geng et al. (2022) [42]	Nölker et al. (2012) [47]	Jin et al. (2015) [48]	Bezerra et al. (2015) [49]
Intervention used	1. Implement a deep convolutional neural network (DCNN) algorithm model for echocardiography. 2. Shengmai injection	Morphine administration and mechanical ventilation using AI algorithms	V-drive robotic system	Remote magnetic navigation (RMN)	Percutaneous coronary intervention, robotic and manual
Control group	1. Routine echocardiography 2. Routine Western medicine treatment	The control group received conventional treatments (sedatives, diuretics, blood vessel expanders, and heart muscle strengtheners).	Not applicable	Not applicable	1509
Sample size (n) intervention/control	Total patients (n = 80) - 40/40	Total (n = 136) - 68/68	A total of 94 patients (23 with paroxysmal atrial fibrillation, 48 with persistent AF, 15 with atrial tachycardias)	Total 313 (PsAF, n = 187, L-PsAF, n= 126)	1673 (n = 164)
Results/findings	After five years of treatment, the observation group showed significant improvements in electrocardiographic parameters, specifically interventricular delay (IVD) and left ventricular electromechanical delay time (LVEMD), while baseline values were similar between groups. Left heart diastolic function was also better in the observation group.	The local gray-scale clustering model (LGSCm) and the fuzzy k-nearest neighbor (FKNN) algorithm showed relatively higher mean absolute deviations (MAD) and maximum mean absolute deviations (max-MAD) as compared to the LGCW model, showing a higher dice matrix value and segmentation accuracy of LGCW.	The study shows that remote manipulation of a circular mapping catheter using the Vdrive system is feasible and safe. Successful procedural outcomes were achieved in all patients, and no adverse event was noted.	This study shows that RMN is safe and effective for achieving pulmonary vein antrum isolation (PVAI) in patients with persistent and long-standing atrial fibrillation (PsAF and L-PsAF). It also reduces the fluoroscopy time during the procedure and does not report significant complications.	The study shows that RA-PCI has a 12.2% incidence of longitudinal geographic miss (LGM) compared to 43.1% in M-PCI.
Salient feature	The research examines explicitly electrocardiographic parameters, left heart diastolic function, and quality of life in 80 patients divided equally into two groups.	This study investigates the effects of morphine administration and mechanical ventilation guided by AI algorithms on heart failure patients.	Evaluation of the V-drive robotic system	Evaluation of remote magnetic navigation (RMN) for catheter ablation	Difference in incidence of LGM in RA-PCI and M-PCI cohorts
Variable measured	Hospitalization status, diagnostic accuracy, left ventricular electromechanical delay time, left ventricular diameter, comprehensive indicator (E/Ea), reverse blood flow velocity (Ar).	Respiratory rate, heart rate, blood pressure, PaO₂, PaCO₂, pH, LVEF, NT-proBNP, total effective rate, dosage of morphine, duration of mechanical ventilation.	Patient characteristics such as age, sex, left atrial diameter, and LVEF. Also, acute ablation efficacy rate, procedure, ablation, and fluoroscopy time.	Patients’ characteristics, such as age, sex, presence of hypertension and heart disease, left atrial volume, pulmonary anatomy, procedure, and fluoroscopy time.	Lesion characteristics include length, diameter, stenosis, minimal lumen diameter, and reference vessel diameter.
Limitations of the study	1. Small sample size	1. Small sample size	1. Study design is non-randomized.	1. Short-term outcomes.	1. It is a retrospective analysis.
	2. Potential deviation in results due to limited content.	2. Differences in baseline characters.	2. Lack of long-term follow-up.	2. No direct comparison	2. Unassessed impact of stent type on longitudinal geographic miss.
		3. Confounding factors		3. Persistent AF unassessed	3. Difference in cohort and limitation of propensity score.
		4. Follow-up period		4. PVAI-CFAE effectiveness	
Duration of study	One and a half years	Two years		6 years (January 2008 to November 2013)	

AI-Powered Chest X-Ray Interpretation for Prompt Heart Failure Identification (ART-IN-HF)

Cardiac dysfunction poses a significant risk because of its nonspecific symptoms, which are particularly challenging to diagnose in its early stages. The utilization of AI for X-ray interpretation offers promising assistance in enhancing early detection, especially in cases of HF with normal ejection fraction [50]. This approach, showcased in the ART-IN-HF study, underscores the potential of AI in improving diagnostic accuracy and enabling timely intervention, ultimately enhancing patient outcomes in HF management. In a study involving 10,100 patients, an AI-powered system identified 183 (1.8%) individuals with raised cardiothoracic ratio and accumulation of pleural fluid on chest X-rays [50]. Among these, 106 underwent diagnostic testing, with 82 (77%) diagnosed with HF according to current guidelines. Among a randomly selected group of 106 patients from the remaining 9,917, nine (8%) were diagnosed with HF. For the diagnosis of HF, the AI system showed a positive predictive value of 77% and a negative predictive value of 91% [50]. Notably, more than half (54.9%) of patients with newly discovered HF had intact ejection fraction. These findings highlight the potential of AI-assisted X-ray interpretation in aiding early detection of HF, particularly HF with normal ejection fraction, which is often challenging to diagnose due to its nonspecific symptoms in early stages [50].

Enhancing Echocardiographic Diagnosis With Deep Convolutional Neural Networks (DCNN)

DCNN-based echocardiographic detection significantly improves diagnostic accuracy compared to routine echocardiography, potentially reducing cardiovascular occurrences in HF patients. This method not only enhances mortality rates but also reduces associated costs. A study utilizing this method highlights improved left heart diastolic function, better quality of life, and enhanced diagnostic accuracy and treatment outcomes associated with the DCNN approach [44]. Several key findings emerged in a study comparing an observation group utilizing echocardiography with a deep learning algorithm to a control group. The observation group showed lower rehospitalization and mortality rates than the control group, but the result was not statistically significant (P > 0.05). However, diagnostic accuracy was notably higher in the observation group. Hospitalization duration and expenses were also reduced in the observation group. Specificity, sensitivity, and overall accuracy of diagnostic methods were statistically higher in the observation arm (P < 0.05). Significant improvements were observed in interventricular delay (IVD) and left ventricular electromechanical delay (LVEMD) parameters in the observation group post treatment (P < 0.05), enhancing overall patient quality of life more significantly than in the control arm (P < 0.05) [44]. This demonstrates how practical deep learning-enhanced echocardiography may be in raising the accuracy of diagnosis, decreasing cardiovascular events, cutting healthcare expenditures, and improving clinical illness detection and treatment [44].

AI-Driven Innovations in Cardiac Imaging

AI innovations in cardiac CT have revolutionized imaging techniques. They offer automated noise reduction and potentially eliminate the need for ICA to assess severe stenosis. AI algorithms automate image analysis, enhancing diagnostic accuracy and reducing healthcare costs [22]. AI CT demonstrates non-inferiority to ICA for left-main/proximal left anterior descending (LAD) stenosis (κ = 0.91 in trials) but remains inferior for distal vessels (κ = 0.67). The current evidence supports selective replacement in low-complexity cases.

Advancing Heart Failure Prediction With Smart Sensors and AI

Recently, studies have explored diverse approaches to forecasting cardiac failure, emphasizing the benefits of both invasive and non-invasive sensors. The sensors, such as wearables, are photoplethysmography (PPG)-based smartwatches detecting fluid retention via circadian rhythm shifts; implantables are pulmonary artery pressure sensors (CardioMEMS) with ML-driven alert systems; and non-invasive sensors are radar-based chest wall motion monitors tracking pulmonary edema. A study investigated improving HF monitoring by integrating smart sensors with AI and the Internet of Things (IoT). By summarizing various strategies for ML in predicting HF, the study provides insights into leveraging technology for more accurate and timely detection, thus improving patient outcomes and management strategies (Table 4) [51].

**Table 4:** Summary of studies evaluating robotic and AI-assisted procedures in cardiology. HT: hemorrhagic transformation; EVT: endovascular treatment; IMA: internal mammary artery; LIMA: left internal mammary artery; SVG: saphenous vein graft; RCS: remote robotic catheter system; MI: mitral isthmus; LVH: left ventricular hypertrophy.

Study ID	van Kranendonk et al. (2019) [52]	Wong et al. (2013) [53]	Kanagaratnam et al. (2008) [54]	Hopkins et al. (2000) [36]
Intervention used	Endovascular treatment (EVT)	Remote robotic catheter system.	Sensi system	Neural network
Control group	256	45 patients	10	100
Sample size (n) intervention/control	478 (n = 222)	90 (n = 45)	20 (n = 10)	317 (n = 217)
Results/findings	Out of 478 patients, 222 experienced HT. HT is divided into four types: 76 patients with hemorrhagic infarction type 1, 71 with HI2, 36 with parenchymal hematoma type 1 (PH1), and 39 with PH2	This study indicates that using a remote robotic catheter system for mitral isthmus ablation is effective and safe. Acute mitral isthmus block was established in 98% of the study participants.	This study successfully conducted remote catheter ablations using the Sensei system, achieving the intended procedural goals without complications or manual manipulation. In addition, the operator's radiation exposure was minimal.	The neural network was quite effective in predicting the presence of left ventricular hypertrophy (LVH) and demonstrated superior performance compared to conventional ECG diagnostic criteria.
Salient feature	Assess the associations between different HT subtypes and functional outcomes in acute ischemic stroke patients with large vessel occlusion.	Assessing the safety and efficacy of the robotic catheter system (RCS) in mitral isthmus (MI) ablation.	Evaluation of a novel remotely controlled steerable guide catheter in various arrhythmias.	The goal is to develop a neural network that can forecast the occurrence of left ventricular hypertrophy (LVH) by utilizing clinical data and ECG readings.
Variable measured	Hemorrhagic transformation types were classified into four groups (HI1, HI2, PH1, PH2), and functional outcome was assessed using the modified Rankin scale (mRS).	Ablation, procedure, and fluoroscopy time. Ablation success rate.	Procedural endpoints include loss of pathway conduction, bidirectional cavotricuspid isthmus block, and pulmonary vein isolation, as well as procedure time and radiation exposure.	ECG data (electrocardiographic data).
Limitations of the study	1. It is a post hoc analysis of a randomized trial, not explicitly designed to determine the association of hemorrhagic transformation (HT) with functional outcome.	1. Small, nonrandomized, single-center study.	1. A small sample size limits the ability to generalize the system's effectiveness and adaptability across various arrhythmia types.	1. This means that the neural network incorrectly identified 35% of the patients without LVH as having LVH (false positives).
	2. Not all 5-day follow-up CT scans were available.	2. The durability of the mitral isthmus block and its impact on medium- to long-term outcomes were not investigated.	2. The robotic system demonstrated longer average procedure times than traditional methods, which is attributed to the learning curve associated with using any new device.	
Duration of study	90 days.	18 months (January 2010 to June 2011)	Not mentioned	Not mentioned
Effect on quality of life	Significant	Nonsignificant	Nonsignificant	Nonsignificant

Role of AI in enhancing early detection of heart failure with telemedicine

Enhancing Remote Monitoring With AI-Powered Wearables

Integrating AI/ML algorithms into remote monitoring systems and wearable devices holds promise for improving workflow and outcomes for HF patients. These algorithms analyze time series data collected through remote monitoring, enabling early detection of impending rehospitalization and enhancing patient management strategies. Recent trials in HF management have shown varied outcomes with different remote monitoring technologies. Smartwatches with PPG detect fluid overload via circadian rhythm shifts, while ECG patches enable seven-day decompensation prediction (AUC = 0.88). The EAGLE Trial by Yao et al., though currently in the protocol stage, is included as the first randomized controlled trial testing continuous PPG/AI alerts for HF decompensation across 12,000 ECGs. Quality of life impacts were specifically measured via KCCQ-12 in wearable studies (TIM-HF2: Δ+8.7 points; BEAT-HF: not assessed), while non-wearable interventions showed nonsignificant effects. This refinement confirms wearables' unique capacity to transform HF management through passive data streams unavailable in telemedicine-only approaches. The telemedical interventional management in heart failure II (TIM-HF2) trial enrolled patients with ejection fraction (LVEF) ≤ 45% and demonstrated better results with online patient management compared to standard medical care [55]. It revealed decreased all-cause mortality (HR: 0.70, 95% CI: 0.50-0.96; p = 0.28) and a smaller percentage of days lost from unscheduled HF-related hospital admissions (HR: 0.80, 95% CI: 0.65-1.00; p = 0.046) when compared to conventional care [55]. Conversely, the BEAT-HF trial concentrated on pre-discharge HF education and telemonitoring but did not demonstrate a significant reduction in 180-day readmission rates (hazard ratio: 1.03, 95% CI: 0.88-1.20; p = 0.74) [55]. This trial underscores the potential of remote monitoring technologies, including wearables and implantable devices, to improve HF management through early detection and personalized treatment strategies facilitated by AI-driven algorithms (Table 5) [56].

**Table 5:** Characteristics of the studies utilizing AI and novel technologies in cardiovascular interventions. AI: artificial intelligence; EHR: electronic health record; EF: ejection fraction; TTE: transthoracic echocardiogram; LVEF: left ventricular ejection fraction; UPDRS: Unified Parkinson's Disease Rating Scale; RKT: robotic kidney transplantation; ACEi: angiotensin-converting enzyme inhibitors; ARB: angiotensin receptor blockers.

Study ID	Budde et al. (1999) [57]	Khan et al. (2013) [58]	Yao et al. (2020) [31]	Mohammadi-Abdar et al. (2016) [59]	Menon et al. (2014) [60]
Intervention used	AI algorithms	Amigo remote catheter system	AI screening tool embedded in the EHR system	Smart exercise bike	GelPOINT device for allograft cooling with ice slush
Control group	Not mentioned	Not mentioned	The control group in the EAGLE trial will receive usual care for heart failure screening.	23	50 consecutive patients who underwent live-donor RKT? Perioperative and postoperative outcomes are reported for the initial 25 recipients who have completed a minimum 6-month follow-up.
Sample size (n) intervention/control	Total 455 patients	206 (n = 194)	12,000 ECGs per arm	47 (n = 24)	90
Results/findings	>95% accuracy in predicting post-procedural complications.	Physicians generally favored the system.	The study concerns the rationale and design of a pragmatic cluster-randomized trial. As the trial has not yet been conducted, no results or findings exist.	The study shows a significant improvement in UPDRS motor III scores in individuals with an average improvement of 13.85%.	Successful robotic transplants (50 patients) with a mean surgery time of 214 minutes; no anastomotic revisions.
	It also identified predictive factors (heart failure class, medication usage)	The Amigo system achieved a high effectiveness rate of 96% in reaching the designated targets, surpassing the pre-specified goal of 80%. When evaluated only by fluoroscopic standards, the success rate was 99%.	-	Significant differences in pedaling metrics	Essential metrics such as average surgery and robotic console use times were 214 and 135 minutes, respectively.
Salient feature	Development and application of a prognostic computer model that utilizes machine learning techniques to predict individual risks of post-procedural complications in patients undergoing coronary intervention.	To assess the efficacy and security of the Amigo system in charting the right side of the heart.	An artificial intelligence (AI) screening tool to detect low ejection fraction (EF).	Participants' motor functions were assessed using the Unified Parkinson's Disease Rating Scale (UPDRS).	Robotic kidney transplantation with regional hypothermia is a safe and effective procedure when performed by a skilled surgical team.
Variable measured	Medical history (heart failure class, prior medications), angiographic findings (stenosis characteristics), procedural factors (adjunctive techniques, ischemia, contrast volume), and medication use (pre-medication	Electrogram amplitudes, threshold, and manipulation time	LVEF, TTE use, prescription of ACEi, ARB, beta blockers, statins, primary care appointments, cardiovascular consults, and implantable cardioverter-defibrillator.	Unified Parkinson's Disease Rating Scale (UPDRS)	Operative and console time. Ischemia times, intraoperative renal surface temperature, blood loss, incision length, postoperative serum creatinine levels, estimated glomerular filtration rate, and postoperative complications.
Limitations of the study	General limitations of such models include generalizability to different populations.	The catheter was used only on the right side of the heart, excluding mapping of the left atrium and left ventricle.	Due to the pragmatic design, clinicians in the intervention group might not always follow Al's TTE recommendations.	Variability in individual responses to the exercise intervention and the unclear mechanism causing improvement in motor function.	Small study size.
	Reliance on data quality and potential overfitting.	The trial is limited to mapping procedures, and no ablation was performed.	Some clinicians may overlook alerts because they do not have time to discuss this issue with patients.	Future research will focus on developing patient-specific algorithms and methods to adapt to changing patient conditions.	No control group.
		-	Others might be concerned about the additional out-of-pocket costs for patients.	Examine the role of proprioceptive input during exercise on motor function.	Short follow-up period (6 months).
Duration of study	Not mentioned	The study enrolled patients between October 2010 and July 2011	Approximately 6-7 months.	40-minute exercise session for 1 week	9 months (January to October 2013)
Effect on quality of life	Indirectly influences patient outcome and quality of life.	Significant by reducing procedure time and complications.	Nonsignificant	Nonsignificant	Nonsignificant

Machine Learning for Tele-Monitoring in Heart Failure

Guidelines suggest early remote monitoring to help promptly identify malfunctioning devices. Additionally, monitoring pulmonary pressure remotely may lower the incidence of HF hospitalizations [61]. These findings underscore the potential of gadgets, remote medical care, and AI in improving cardiac dysfunction outcomes by facilitating timely interventions and reducing hospitalizations [62]. If incorporated into HF telemonitoring, ML can be a game-changer. Lee et al. (2011) deployed a home-based mobile rehabilitation system using wireless ECG monitoring to track heart and respiratory rates. Their ML-derived respiratory rate correlated with airflow measurements at 80.55% accuracy, while heart rate measurements achieved 98.5% correlation with clinical recorders. Oikonomou et al. (2022) applied an XGBoost algorithm to predict hospitalization risk using systolic/diastolic blood pressure data from 4,678 patients. The model stratified intensive treatment benefits, showing a median hazard ratio of 0.63 for major cardiovascular events. Mada et al. (2016) used speckle-tracking echocardiography (start systolic index (SSI) and peak longitudinal displacement (PLD) parameters) to identify responders to cardiac resynchronization therapy (CRT). Validated in 100 patients, ML achieved accuracy comparable to expert readers, distinguishing CRT efficacy in HF with reduced ejection fraction. These studies demonstrate ML’s ability to discern decompensated states, differentiate HF subtypes, and predict hospitalization risk using daily metrics, enabling timely interventions.

A study showcased the integration of ML methods with tele-monitoring to discern compensated and decompensated states in HF patients. They also recognize different HF types with varying clinical responses. Additionally, ML can detect hospitalization risk using daily data like blood pressure and weight in patients with HF, achieving high accuracy. ML systems can also anticipate responses to CRT [63]. This approach offers potential advancements in the management of HF, enabling timely interventions and personalized care based on patients' real-time physiological data.

AI's Contribution to Enhancing Heart Failure Detection

AI has recently been incorporated into developing a decision-making framework for early identification of HF in intermediate or indeterminate cases by facilitating timely diagnosis and intervention [64]. Leveraging AI in decision-making processes is a promising avenue in cardiovascular medicine by improving the overall prognosis and care of HF patients.

A study employed ECG data to diagnose CHF using an 11-layer DCNN [65]. The CNN model, requiring minimal pre-processing and no engineered features, achieved high accuracy across different datasets, with one set reaching 98.97% sensitivity and 99.01% specificity [65]. This model aims to provide cardiologists with faster and more objective ECG signal interpretations. In another study, a different algorithm, named a deep learning (DL) algorithm, was used for identifying HF based on ECGs from 22,765 patients across two hospitals. The DL algorithm outperformed traditional ML methods in accuracy and validated its effectiveness in HF detection using ECG features [65]. The study by Misumi et al. (2021) used ML (unspecified model) on acoustic spectra from left ventricular assist device (LVAD) sounds to predict aortic regurgitation. The model achieved 91% accuracy and an AUC of 0.73, validated over 227 ± 160 days post implant. Both studies used derivation-test cohorts and cross-dataset validation. Performance metrics included AUC, sensitivity, specificity, and F1-scores (Table 6).

**Table 6:** Effects of combined therapy on heart failure outcomes. EDR: ECG-derived respiration; AR: aortic regurgitation; LVAD: left ventricular assist device; Pmus: inspiratory muscle pressure; Eadi: electrical activity of the diaphragm; Ccw: compliance of the chest wall; SSI: start systolic index; PLD: peak longitudinal displacement; CRT: cardiac resynchronization therapy; MACE: major adverse cardiovascular event.

Study ID	Lee et al. (2011) [66]	Dehais et al. (2011) [67]	Misumi et al. (2021) [68]	Silva et al. (2023) [69]	Oikonomou et al. (2022) [70]	Mada et al. (2016) [71]
Intervention used	A home-based mobile rehabilitation system includes a wireless ECG Holter.	Cognitive countermeasures to mitigate attentional tunneling in uncrewed vehicle operations (n = 11).	Machine learning was applied to acoustic spectra from LVAD sound signals to predict significant aortic valve regurgitation (AR).	Inspiratory muscle pressure (Pmus) waveform.	Extreme gradient boosting algorithm (XGBoost).	The EchoPAC prerelease software. A subgroup of 25 subjects was randomly sampled for deriving cutoff values for the tested parameters (derivation group (DER)).
Control group	Not given	12	219	49	n = 4683	100 subjects were used to test the performance of the new methods (test group (TEST)).
Sample size (n) intervention/control	15	23 (n = 11)	245 (n = 26)	98 (n = 49)	9361 (n = 4678)	Total 125 (randomly selected, n = 25, 100 test group)
Results/findings	1. Heart rate measurement correlates highly (98.5%) with a commercial clinical recorder.	1. The cognitive countermeasure group showed enhanced attentional abilities compared to the control group.	1. Machine learning predicted significant AR with 91% accuracy and an area under the curve (AUC) of 0.73.	1. The display of the Pmus waveform improved healthcare professionals' ability to recognize patient-ventilator asynchronies.	1. Intensive treatment was generally favored in reducing major cardiovascular events (MACE) in SPRINT participants with a median individualized hazard ratio of 0.63.	1. New speckle-tracking-based quantitative parameters, SSI and PLD, effectively identify CRT responders with accuracy and reproducibility comparable to visual analysis by expert readers.
	2. The derived respiratory rate from ECG has up to an 80.55% correlation with an airflow meter.	2. Ten out of 11 participants in the countermeasure group made appropriate decisions during a battery failure event.	-	-	2. Additionally, when applied to the ACCORD BP trial, it shows a lower actual MACE risk compared to standard treatment.	-
Salient feature	Development of a mobile home-based cardiopulmonary rehabilitation system, providing real-time exercise feedback and integrating an arrhythmia detection system.	The study demonstrates the effectiveness of cognitive countermeasures in enhancing situational awareness and decision-making during automation conflicts.	Utilizing machine learning to analyze acoustic spectra from LVAD sounds for AR prediction represents a novel approach in the field.	Improve healthcare providers' ability to identify patient-ventilator asynchronies.	Development of a machine learning-based tool using data from SPRINT and ACCORD BP trials to define the cardiovascular benefit of intensive control of systolic blood pressure.	Using new echocardiographic parameters such as start systolic index (SSI) and peak longitudinal displacement (PLD), identify responders to cardiac resynchronization therapy (CRT).
Variable measured	Heart rate and respiration rate	Attentional focus and decision-making in response to automation conflicts, assessed via heart rate and eye tracking.	Acoustic spectra features from LVAD sound signals to predict AR.	Pressure and flow waveform	Systolic blood pressure and diastolic blood pressure	Start systolic index (SSI) and peak longitudinal displacement (PLD).
Duration of study	Not mentioned	Not mentioned	The patients were followed for a mean time of 227 ± 160 days post-LVAD implantation.	Not mentioned	Not mentioned	Immediately before and 6 to 12 months after CRT implantation

In a separate study, electronic health records and DL techniques, specifically GANs, were used to predict treatment effects in HF patients. This approach demonstrated superior performance compared to benchmark models, indicating DL's potential to automate healthcare processes and support clinicians in early HF risk identification and treatment planning [65].

## Conclusions

In conclusion, this narrative review sought to highlight the role of AI in improving the quality of life of patients living with HF through an analysis of a broad range of studies. AI significantly advances HF management through enhanced diagnostics, telemonitoring precision, and personalized therapies, demonstrated by ECG-based CNNs achieving 99% specificity, XGBoost algorithms reducing hospitalizations via remote data, and speckle-tracking echocardiography optimizing CRT responses. These successes provide a validated foundation for extending AI benefits to other chronic conditions such as diabetes and chronic obstructive pulmonary disease, where similar data-driven personalization can improve outcomes. AI also strengthens patient-clinician communication by transforming complex analytics into visual, interpretable insights for shared decision-making. However, key challenges such as technical, ethical, and equity challenges require resolution by minimizing false alerts via adaptive thresholds without compromising sensitivity, addressing algorithmic bias, and establishing accountability frameworks, prioritizing socioeconomic, geographic, and ethnic diversity in trials to prevent outcome disparities, respectively.

The future progress demands multicenter trials across diverse populations, standardized validation for cross-institutional model reliability, EHR-integrated AI enabling real-time comorbidity prediction (such as atrial fibrillation and renal dysfunction), and interdisciplinary collaboration uniting clinicians, data scientists, and policymakers. While some studies reviewed did not directly apply AI, their insights remain valuable for scalable solutions. Proactive attention to these priorities will ensure AI’s ethical, equitable integration across healthcare.
